# RETRACTED: Procópio de Oliveira et al. Potential Protective Role of Melatonin in Benign Mammary Cells Reprogrammed by Extracellular Vesicles from Malignant Cells. *Biomedicines* 2023, *11*, 2837

**DOI:** 10.3390/biomedicines13051029

**Published:** 2025-04-24

**Authors:** Caroline Procópio de Oliveira, Barbara Maria Frigieri, Heidge Fukumasu, Luiz Gustavo de Almeida Chuffa, Adriana Alonso Novais, Debora Aparecida Pires de Campos Zuccari

**Affiliations:** 1 Cancer Molecular Research Laboratory (LIMC), Faculdade de Medicina de São José do Rio Preto—FAMERP, Av. Brigadeiro Faria Lima, São José do Rio Preto 15090-000, SP, Brazil; carolineprocopio_oliveira@hotmail.com (C.P.d.O.); barbara.frigieri@unesp.br (B.M.F.); 2 Postgraduate Program in Health Sciences, Faculdade de Medicina de São José do Rio Preto—FAMERP, Av. Brigadeiro Faria Lima, 5416, São José do Rio Preto 15090-000, SP, Brazil; 3 Institute of Biosciences, Letters and Exact Sciences (IBILCE) UNESP, São José do Rio Preto 15054-000, SP, Brazil; 4 Faculty of Animal Science and Food Engineering, University of São Paulo, Pirassununga 13635-900, SP, Brazil; fukumasu@usp.br; 5 Department of Structural and Functional Biology, Institute of Biosciences, São Paulo State University (UNESP), Botucatu 18618-689, SP, Brazil; luiz-gustavo.chuffa@unesp.br; 6 Institute of Health Sciences (ICS), Federal University of Mato Grosso (UFMT), Sinop 78550-728, RS, Brazil; aanovais@terra.com.br

The journal retracts the article, “Potential protective role of melatonin in benign mammary cells reprogrammed by extracellular vesicles from malignant cells” [1].

Following publication, concerns were brought to the attention of the publisher regarding duplicate figures between this article [1] and three other publications [2,3,4], produced by different authorship groups.

Adhering to our complaint procedure, an investigation was conducted by the Editorial Office and Editorial Board that confirmed duplications between panels in Figure 4 in [1]; Figure 3A,B in [2]; Figure 5E in [3]; and Figure 7A in [4], representing different experimental conditions. While the authors cooperated with the Editorial Office during the investigation, they were unable to satisfactorily explain the overlap of figures nor provide appropriate raw material for Editorial Board evaluation. Consequently, the Editorial Board has lost confidence in the reliability of the findings and decided to retract this publication [1], as per MDPI’s retraction policy (https://www.mdpi.com/ethics#_bookmark30).

This retraction was approved by the Editor-in-Chief of the journal *Biomedicines.*

The authors agreed to this retraction.
